# Underrecognized Interaction Between PICO Dressings and Pacemakers: A Case From the ICU

**DOI:** 10.1155/cria/2189554

**Published:** 2026-08-04

**Authors:** Reginald Caldecott, John Laffey

**Affiliations:** ^1^ Department of Anaesthesia and Intensive Care Medicine, University Hospital Galway, Galway, Ireland, hse.ie; ^2^ Department of Anaesthesia, School of Medicine, University of Galway, Galway, Ireland, universityofgalway.ie

**Keywords:** asynchronous pacing, cardiac implantable electronic device, ICU safety, magnet mode, negative pressure wound therapy, pacemaker, PICO dressing

## Abstract

**Background:**

Modern pacemakers incorporate a magnet‐responsive switch that, on exposure to a sufficient static magnetic field, converts the device to fixed‐rate asynchronous (magnet‐mode) pacing. This is designed behaviour rather than malfunction. Interactions between this feature and magnet‐containing medical equipment are underrecognised. The PICO 7 negative pressure wound therapy (NPWT) system contains a magnet in its pump unit, which the manufacturer states can interfere with nearby pacemakers.

**Case Presentation:**

An 81‐year‐old man was admitted to the High Dependency Unit after right hemicolectomy. He had atrial fibrillation with a slow ventricular response and a permanent pacemaker programmed VVI at 60 beats per minute. Overnight, telemetry showed a sustained paced rate of 100 beats per minute; he was asymptomatic. The PICO 7 pump unit was found resting on his chest, over the pacemaker generator. Repositioning the pump to the foot of the bed returned the paced rate to 60 beats per minute immediately, and device interrogation the following day confirmed normal pacemaker function.

**Discussion:**

The fixed rate of 100 beats per minute, its dependence on pump position and immediate resolution on removal are characteristic of magnet‐mode activation rather than generic electromagnetic interference. Although the patient was asymptomatic, a sustained rate of 100 beats per minute carried a risk of myocardial ischaemia and heart failure decompensation given his cardiac history. Recognition and a simple bedside intervention resolved the episode.

**Conclusion:**

Magnet‐containing medical equipment placed near a cardiac implantable electronic device can trigger magnet‐mode pacing. The risk depends on the position of the magnet‐bearing component, not on the wound site. An unexplained change in a paced rate should prompt early consideration of a nearby magnetic source, because the remedy may be immediate and at the bedside.

## 1. Introduction

Pacemakers regulate cardiac rhythm in patients with bradyarrhythmias and conduction disease [1]. They incorporate a magnet‐responsive switch: When a static magnetic field of sufficient strength is applied over the device, the switch closes and the pacemaker reverts to asynchronous, fixed‐rate pacing at a manufacturer‐specific magnet rate [2]. This is an intended safety and testing feature rather than a fault. When such a field is applied unintentionally, the same feature can produce an unexpected change in paced rate, which may matter in critically ill patients.

Unintended magnet‐mode activation from equipment placed near a device has been reported, and modern consumer devices containing compact magnet arrays, such as smartphones with magnetic charging accessories, can trigger the switch at close range [3]. The PICO 7 negative pressure wound therapy system contains a magnet in its pump unit; the manufacturer states that it can interfere with pacemakers and should be kept at least 10 cm from such devices [4]. Reports specifically describing interaction between an NPWT system and a pacemaker remain sparse.

We describe a case in the intensive care setting in which a PICO 7 pump, used for wound management [5], was associated with a change in a patient’s paced rate from 60 to 100 beats per minute. The episode illustrates the importance of recognising magnet‐mode activation from equipment not traditionally regarded as hazardous to pacemakers. We aim to raise clinician awareness of this interaction, to clarify the underlying mechanism and to suggest practical measures to prevent recurrence.

### 1.1. Patient and Contextual Information

An 81‐year‐old man was admitted to the High Dependency Unit after right hemicolectomy for suspected abdominal malignancy.

### 1.2. Cardiac History

Atrial fibrillation with slow ventricular response is managed with a permanent pacemaker programmed VVI at 60 beats per minute; chronic congestive heart failure with raised preoperative proBNP; angina on exertion; and moderate carotid stenosis.

### 1.3. Other History

Other histories are iron deficiency anaemia, benign prostatic hyperplasia, and a clinical frailty score of 4 with good preoperative functional status.

The hemicolectomy was uneventful. Postoperatively, he was admitted to the High Dependency Unit for continuous telemetry monitoring, and his early postoperative course was stable.

### 1.4. Context of the Incident

A PICO 7 dressing was applied to the midline abdominal incision to support wound healing. Overnight, nursing staff on the High Dependency Unit noted a sustained rise in heart rate, from the pacemaker’s programmed 60 to 100 beats per minute, on continuous telemetry. The patient was asymptomatic when reviewed by the on‐call team.

The dressing itself was over the abdomen, a site not intuitively associated with pacemaker interference. The unexplained change in a stable patient prompted further assessment.

### 1.5. Assessment, Optimisation and Interventions

#### 1.5.1. Assessment

The patient was asymptomatic, without chest pain, palpitations or dizziness. The electrocardiogram showed a pacing spike preceding each QRS complex at a fixed rate of 100 beats per minute, confirming that the pacemaker was pacing at the elevated rate. The on‐call cardiology team was consulted; as the patient was asymptomatic and haemodynamically stable, they advised no immediate intervention, with electrophysiology review planned for the following morning.

#### 1.5.2. Intervention

On further review, the PICO 7 pump unit, which generates the negative pressure, was found resting directly on the patient’s chest, over the pacemaker generator. The pump was moved to the foot of the bed, and the paced rate returned to the programmed 60 beats per minute immediately.

The following day, the electrophysiology service interrogated the pacemaker and confirmed normal device function. The patient remained haemodynamically stable throughout, continued an uneventful recovery and was discharged to ward‐based care the following day.

## 2. Discussion

The features of this episode, a clean fixed rate of exactly 100 beats per minute, dependence on the position of the pump and immediate resolution when the pump was removed, are characteristic of magnet‐mode activation rather than generic electromagnetic interference. Pacemakers contain a magnetically activated switch (a reed switch or Hall‐effect sensor); a static magnetic field above the switch threshold, applied close to the generator, closes the switch, and the device paces asynchronously at a fixed manufacturer‐specific magnet rate [2]. A magnet rate of 100 beats per minute corresponds to Boston Scientific and Abbott devices, whereas Medtronic devices use 85 beats per minute [6], so the observed rate is consistent with a Boston Scientific or Abbott device; the exact device could not be confirmed (see Limitations). By contrast, true electromagnetic interference typically causes oversensing with inappropriate inhibition or noise reversion, not a clean sustained fixed rate. The PICO 7 pump unit contains a magnet, and the manufacturer states that it can interfere with pacemakers and should be kept at least 10 cm away [4]; resting on the chest over the generator, its field was sufficient to activate magnet mode.

We regard the association as temporal, supported by a positive dechallenge: Repositioning the pump normalised the rate immediately. Deliberate re‐exposure (rechallenge) was not performed. Alternative explanations are unlikely. A rate‐response (activity) sensor was excluded because the device was programmed VVI without rate modulation, and the response tracked pump position rather than patient activity. Atrial tracking is not possible in a single‐chamber VVI device. Oversensing would be expected to inhibit pacing or cause noise reversion rather than produce sustained fixed‐rate pacing, and genuine pacing spikes preceded each QRS complex, making artefact unlikely. Accordingly, we describe the change as temporally associated with likely magnet‐mediated activation rather than as definitively caused by the pump.

Although the patient was asymptomatic, a sustained rate of 100 beats per minute in a patient with atrial fibrillation, congestive heart failure and carotid stenosis could increase myocardial oxygen demand and reduce cardiac output, with a risk of ischaemia [7, 8] or heart failure decompensation in the perioperative period.

Magnet‐mode activation by nearby magnetic sources is recognised. Consumer smartphones with magnetic charging arrays, for example, have been shown to activate the magnet switch of implanted cardiac devices at close range [3], illustrating the same mechanism. The present case extends this to a bedside medical device, the NPWT pump.

Two independent recommendations converge on separation distance: the British Heart Rhythm Society advises keeping sources of interference at least 15 cm from a cardiac implantable electronic device [9], and the PICO 7 manufacturer advises at least 10 cm from other medical devices [4]. In this case, the pump lay directly on the chest, well within both. This guidance fails silently at the bedside for a specific reason: The magnet is contained in the pump unit rather than the dressing, staff applying NPWT are generally unaware that the pump contains a magnet, there is no bedside prompt for device proximity and the pump tends to be positioned for tubing reach and convenience rather than device safety.

Risk, therefore, attaches to the position of the magnet‐bearing pump unit, not to the wound site: Although the dressing was abdominal, the pump on the chest produced a thoracic effect. Any patient with a cardiac device and a magnet‐containing pump that can rest near the generator is at risk, regardless of wound location. Practical mitigation is simple: secure the pump unit away from the device, incorporate a pump‐positioning check for patients with cardiac devices and flag the magnet at the time of NPWT application. Review was reasonably deferred overnight because the patient was asymptomatic and stable; the wider lesson is that an unexplained change in a paced rate should prompt early consideration of a nearby magnetic source, since the remedy may be immediate and at the bedside.

### 2.1. Key Points

1. A clean, fixed, manufacturer‐specific paced rate that resolves when a nearby object is removed indicates magnet‐mode activation, not generic electromagnetic interference.
2. The risk follows the position of the magnet‐bearing component, here the NPWT pump unit, not the wound site.
3. We consider a nearby magnetic source early when a paced rate changes unexpectedly; the correction may be immediate and at the bedside.

### 2.2. Limitations

This is a single case. The specific pacemaker, its manufacturer, model, lead configuration and programming logs could not be retrieved and are, therefore, unavailable; the magnet‐mode interpretation rests on the fixed, manufacturer‐consistent rate, the spatial dependence on pump position and the positive dechallenge, rather than on interrogation during the episode. The device was not interrogated at the moment of the event, rechallenge was not performed, and the magnetic field of the pump was not measured in situ.

## Funding

No funding was received for this manuscript.

## Conflicts of Interest

The authors declare no conflicts of interest.

## Patient Perspective

From the patient’s perspective, the episode was entirely asymptomatic. He was unaware of any change in his heart rate, which was detected by nursing staff on continuous telemetry and reported no chest pain, palpitations, breathlessness or distress at any point. On explanation by the clinical team, he was reassured that the cause had been identified promptly and resolved by a simple change in the position of the wound‐therapy pump, and his recovery was otherwise uneventful.

## Data Availability

Data sharing is not applicable to this article as no datasets were generated or analysed during the current study.
